# Maud Menten: Pioneering Pediatric-Perinatal Pathologist, Clinician-Scientist, and “the Most Wonderful Human Being in the World”

**DOI:** 10.1177/10935266231202934

**Published:** 2023-11-25

**Authors:** James R. Wright

**Affiliations:** 1 Department of Pathology & Laboratory Medicine, University of Calgary, Calgary, AB, Canada

**Keywords:** history of pathology, Maud Menten, Michaelis-Menten equation, physiological surgery, Pittsburgh Children’s Hospital, women in science

## Abstract

Maud Menten was born and raised in remote regions of Canada. She obtained her MB/MD at the University of Toronto (1907/1911) and her PhD in biochemistry at the University of Chicago (1916). From 1907 to 1916, she trained at the Rockefeller Institute for Medical Research, the New York Infirmary for Women and Children, Western Reserve University in Cleveland, the Berlin Municipal Hospital in Germany, and the Barnard Free Skin and Cancer Hospital in St Louis. In 1916, she was appointed as pathologist at the Elizabeth Steel Magee Hospital, a charitable maternity hospital in Pittsburgh. She received a faculty appointment at the University of Pittsburgh (1918) and was appointed pathologist at Pittsburgh Children’s Hospital (1926). In addition to being one of the first woman academic pathologists, she was likely the first perinatal, the second pediatric-perinatal, and the fourth pediatric pathologist to practice in North America. The importance of Menten’s overall scientific contributions place her in the very upper echelon of 20th century pathologists. Her enzyme kinetic work resulted in the Michaelis-Menten equation, and her work in George Crile’s laboratory in Cleveland provided a physiological basis for improved surgical outcomes. Her work in Pittsburgh was equally innovative, including initiating the field of enzyme histochemistry.

In the age of the Internet, Maud Menten (1879–1960; Figure 1) has gained some degree of fame which had evaded her throughout her lifetime as well as most of the 20th century; she is currently the topic of many articles available online, blogs, etc. focused on her lack of recognition and as a prime example of the unfair treatment of early 20th century women in science; however, few of these sources provide much detail and most rehash the same few talking points. Much of this publicity was initiated by a charming article by Rebecca Skloot entitled “Some called her Miss Menten,” which appeared in the University of Pittsburgh (Pitt) *Pitt Med Magazine*, in October 2000.^1^ Skloot, who was at the time working as a writer for *Pitt Med* while drafting her award-winning book *The Immortal Life of Henrietta Lacks* (i.e., the standard history of the HeLa cell line),^2^ was a natural author for a paper on Menten as she is a Pitt alumnus and possesses a near-lifelong passion for “women forgotten by science.”^3^

**Figure 1.**
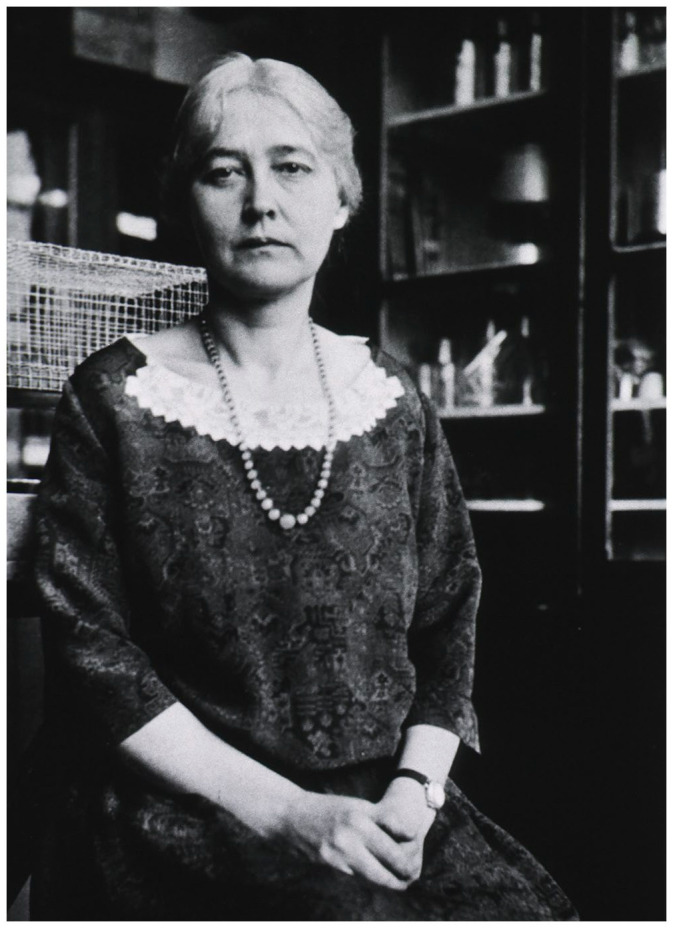
Maud Menten, MD, PhD in her University of Pittsburgh laboratory. National Library of Medicine Digital Collection. https://collections.nlm.nih.gov/catalog/nlm:nlmuid-101423399-img.

Unfortunately, no single source, either online or in print, does justice to this pioneering woman pediatric pathologist clinician-scientist, and she is little known by our profession. As a Canadian MD, PhD who spent most of her working career in the US, Menten has apparently fallen between the “cracks at the 49th parallel (north),” as standard historical reference books on early women medical scientists on both sides of the Canadian^4,5^-American^6-10^ border fail to mention her. However, because of her primary role in developing the Michaelis-Menten equation, she is better recognized by biochemists interested in the history of enzymology (see below), though she is barely mentioned in Louis Rosenfeld’s *Four Centuries of Clinical Chemistry*.^11^ Sadly, she does not appear in Jan van den Tweel’s *Pioneers in Pathology*.^12^

The importance of Menten’s overall scientific contributions to laboratory medicine should place her in the very upper echelon of 20th century pathologists, regardless of gender or area of specialization. However, other than her obituary in *Nature*,^13^ a short paper in *Perspectives in Pediatric Pathology* written by one of her devoted former trainees,^14^ a brief entry in a biographical dictionary of women in science,^15^ and a more substantial entry in *Dictionary of Canadian Biography*,^16^ there is limited information about her impressive life and career in the scholarly historical literature.

## Pioneering Pediatric-Perinatal Pathologist

Maud Menten was1 of the 4 earliest fulltime pediatric pathologists in North America. All 4 of these pioneers predated the better recognized founders of pediatric-perinatal pathology: Sidney Farber (1903–1973) at Boston Children’s Hospital in 1929^17^ and Edith Potter (1901–1993) at Chicago’s Lying-in Hospital in 1934^18^ as well as the cohort of 35 pediatric pathologists who formed the Pediatric Pathology Club, the predecessor of the Society for Pediatric Pathology, in 1966.^19-21^

Martha Wollstein (1868–1939), who was appointed as pathologist at Babies Hospital in New York City in 1891, was the first North American pediatric pathologist.^22,23^ Ward Thomas Burdick (1878–1928) was likely the second, as he was appointed laboratory director when the new Denver Children’s Hospital opened its doors in 1917; however, Burdick is primarily known as a co-founder of the American Society for Clinical Pathology.^24,25^ There is no evidence that Burdick ever held an academic appointment, and his limited publication record does not demonstrate a special interest in pediatric pathology but rather is narrowly focused on organizing the pathology profession in America.^24^

Little information is available related to Isaac Huber Erb (1886–1965), who was appointed as a pathologist at Toronto’s Hospital for Sick Children in 1919 and director of pathology and bacteriology in 1921; however, his pediatric pathology job was not fulltime, as he simultaneously served as consulting pathologist for the provincial attorney general’s office, and he had an extensive clinical private practice treating allergies. He was appointed an assistant professor of pathology at the University of Toronto (UofT) late in his career. He is likely the third North American children’s hospital-based pathologist.^26,27^

Where to place Maud Menten in this list is more complicated depending upon whether one “lumps” pediatric and perinatal pathology together. She obtained her MB and MD medical degrees at the UofT in 1907 and 1911, respectively, and her PhD in biochemistry at the University of Chicago in 1916. Her first faculty appointment was as a fulltime demonstrator in pathology at the Pitt School of Medicine in 1918; based upon her research productivity as an experimental pathologist, she was promoted to assistant professor in 1923 and associate professor in 1926, at which time she was appointed as the pathologist at Children’s Hospital of Pittsburgh; Menten served as its director of laboratories until her retirement in 1950. While none of her biographical sources account for her time between obtaining her PhD and her appointment as demonstrator, Menten’s entry in *American Men of Science* lists her as the pathologist at the Elizabeth Steele Magee Hospital in Pittsburgh from 1916–1918.^28^ Menten published at least 1 paper “from the Pathological Laboratories of the Elizabeth Steel Magee Hospital.^29^ The Elizabeth Steel Magee Hospital was primarily a charitable maternity hospital established in 1911 that merged with Pittsburgh Woman’s Hospital in 1962 to become Magee-Womens Hospital.^30,31^ While the sufficiency of her chemistry and bacteriology skills to function as a pathologist would have been unassailable by that time in her research career, I have found no source documenting any formal anatomical pathology (AP) training. While she had used histology in her research, most of her AP skills appear to be self taught while performing perinatal autopsies. In summary, Menten was almost certainly the first perinatal pathologist, the second pediatric-perinatal pathologist, the fourth children’s hospital-based pediatric pathologist, the second pediatric pathologist with an academic appointment, and one of the first woman academic pathologists with full scope hospital practice in North America.

Perhaps not surprisingly because of the rarity of women in pathology in the early 20^th^ century,^32^ Menten met Wollstein early in Menten’s career, as was incidentally reported in a brief public interest story in a Pittsburgh newspaper in 1908. The article simply announced that 3 women had been appointed as fellows at the Rockefeller Institute for Medical Research in New York City. Two of these were “Miss Maud L. Menten and Miss Wollstein.”^33^ Since Wollstein had already been productively working at the Institute for over 5 years and had already published several independent papers in its highly prestigious *Journal of Experimental Medicine*, it was demeaning for Wollstein that, because she was a woman, she was ineligible for a permanent scientific position and needed to be reappointed annually as a fellow.^22,23^ Furthermore, it was likely awkward as a pathologist with 17 years experience as director of a hospital laboratory to have the same job title as Menten, who in 1908 was simply a promising research novice. Wollstein was highly outspoken about gender inequality at the Institute, and she eventually resigned after insisting unsuccessfully on being treated the same as its male scientists.^22,23^ It is safe to assume that Menten was well aware that Wollstein was the only woman practicing pathology at any teaching hospital in North America^32^; knowing that Wollstein was a clinician-scientist pediatric pathologist may have influenced Menten’s future career choices and guided her toward pediatric pathology.

Following in Wollstein’s footsteps, Menten became one of the earliest North American women providing anatomic and clinical pathology services at an academic hospital. It is worth noting that the job descriptions of medical school-affiliated women “pathologists” in the early 20^th^ century, with the exception of Wollstein, had been limited to bacteriology, experimental pathology, or teaching pathology to medical students.^32^ Prominent early 20^th^ century women academic pathologists with extensive AP training from top centers such as Maude Abbott (1868–1940), Lydia DeWitt (1859–1928), Dorothy Reed (1874–1964), and Alice Hami-lton (1869–1970) were barred from providing AP clinical services.^32^ Even at non-academic hospitals, women pathologists were exceedingly rare during the first few decades of the 20th century; 2 of the earliest worked sequentially in a small frontier western Canadian town.^34^

## Early Life and Education

**Figure 2.**
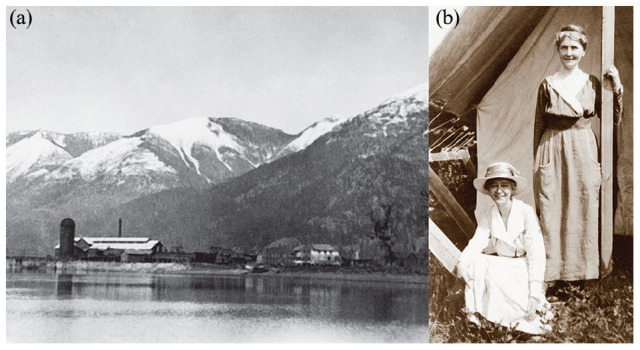
a. (left) Harrison Mills, British Columbia, circa 1902–1903. From left to right, railroad bridge, beehive burner, Rat Portage Lumber Mill, housing, and Menten Store/Post Office. Credit: Kilby Historic Site, image KGSM1975.019.030. b. (right) Maud (sitting) and Emma (standing) Menten outside of a canvas tent. Date unknown. Credit: Kilby Historic Site, image KGSM1984.025.008.

Maud Menten was born on March 20, 1879 in Lambton County, Ontario, Canada. She was the daughter of Charles William Menten (1844–1903) and Emma (neé Trusler) Menten (1859–1932). She had a younger brother, Robert (Bob) Clarence Menten (1881–1944). Having heard of exciting opportunities due to the westward expansion of the Canadian Pacific Railroad (CPR), the Menten family moved in the autumn of 1889 to Harrison Mills, British Columbia (BC), a small logging town on Harrison Bay at the convergence of the Harrison and Fraser Rivers in southern BC (Figure 2(a)). At the time, Emma and the mill owner’s wife were the only 2 women of settler origin in the small town.^35^ Harrison Bay is the traditional home of both the Sq´éwlets and Sts´ailes indigenous peoples, who speak different dialects of Halq´eméylem language. Maud grew up playing with indigenous children and learned to speak Halq´eméylem, for which she retained lifelong fluency.^16,35^ Considerable detail is available on both Harrison Mills and the Menten family through the Kilby Historic Site at Harrison Mills (Kilby Heritage Society, http://www.kilby.ca).^35^

William became the town’s postmaster in 1890 and was a river boat pilot ferrying passengers between the larger city of Chilliwack, BC, which did not have CPR service, and Harrison Mills, which did. Emma was owner/operator of a hotel and a general store (Figure 2(a) and (b)). She provided early home-schooling for her children, as there was no school in the town until 1901. In 1892, a 1-room schoolhouse opened on Fairfield Island near Chilliwack; Maud and Bob crossed the river by canoe and then traveled another 2½ miles either on foot or by pony to attend classes. In 1894, Maud passed the high school entrance exam and then attended high school in Chilliwack for 3 years, canoeing the Fraser River ~3 miles each direction. She graduated with honor roll distinction in 1897. Beginning in August 1897, Maud taught for 3 years at a small rural school in nearby Camp Slough, BC, earning $50/month toward future higher education.^16,35^ Camp Slough is across the Fraser River from Harrison Mills.

**Figure 3.**
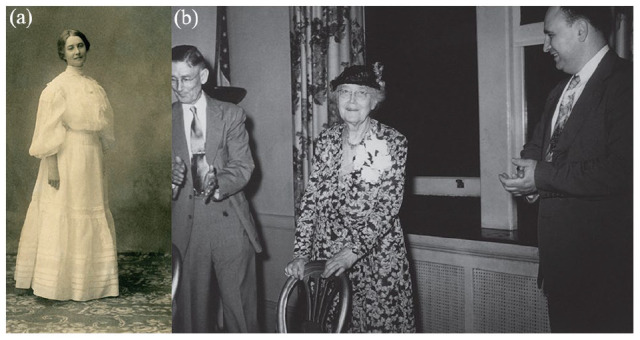
a. (left) Maud Menten at the beginning of her career, likely while a student in Toronto. Credit: Kilby Historic Site, image KGSM1975.022.036. b. (right) Maud Menten at University of Pittsburgh retirement party with her department head George Lacey (left) and colleague Aaron Stock (right). Credit: Kilby Historic Site, image KGSM1984.025.016.

Throughout her later professional life, Menten’s colleagues always emphasized the seeming incongruity of how such a petite, ultrafeminine woman (Figure 3(a) and (b)) could in reality be so tough. Likely, this characteristic was forged in her youth going to and from schools.

### Medical Education and a Brief Chronological Overview of Her Research Training

In 1901, Menten enrolled at UofT, completing a BA in natural science and English with honors in 1904. In 1904–1905, she worked as an assistant demonstrator in physiology for Professor Archibald Byron Macallum (1858–1934)^36^ while studying for her MB.^13,15,16^ They published a paper describing the distribution of chlorides in nerve cells and fibers in 1906.^37^ Also in 1906, Menten was elected into the UofT Sigma Chapter of Kappa Alpha Theta sorority.^38^ She graduated with her MB in 1907.^13,16^ Clearly, her lifelong career in medical research began at UofT, and Macallum helped her obtain future research positions; however, UofT medical historian Edward Shorter warns against his institution claiming too much credit for her future success:

History is not served by the creation of myths, and the notion that Maud Menten, an important pathologist and enzymologist, somehow achieved greatness while at the University of Toronto is a myth. She. . . graduated with her MB from UofT in 1907, one of what were by then numerous female graduates. She gained an MD from U of T in 1911. . . [and then moved on] (p. 548).^39^

Nevertheless, several years later, she published her first sole-authored paper based upon work she had done earlier in Macallum’s laboratory.^40^

In 1907–1908, Menten worked as a pathology research fellow with Simon Flexner (1863–1946) and James Wesley Jobling (1876–1961) at the Rockefeller Institute for Medical Research.^13,15,16^ They published a monograph entitled *Tumors of Animals* in 1910; Menten’s contribution described the effects of radium bromide on the Flexner-Jobling transplantable rat tumor.^41^ Her work was begun only 9 years after radium bromide’s discovery by Pierre and Marie Curie; this was also the first monograph published by the Institute.

Menten next worked as an intern at the New York Infirmary for Women and Children; while no sources provides dates, Ogilvie and Harvey report this was a 1 year position.^15^ New York Infirmary for Women and Children was a hospital for women in Lower Manhattan that was run by women; it was established by Elizabeth Blackwell (1821–1910), the first woman in the US to graduate from medical school.^42,43^ This position is not mentioned in Menten’s *Nature* obituary^13^ nor is Menten mentioned in a recent history of Blackwell, her sister, and their institutions.^43^ Unfortunately, Annie Sturgis Daniel’s serialized history of both the Infirmary and its associated medical school narrowly misses the time period of Menten’s position and so is not helpful.^44^

Menten was a lifelong “multitasker,” which makes all reports of her biographical information confusing; further complicating matters, ambitious aspiring women medical scientists in the early 20^th^ century often gained much of their research experience by doing informal volunteer work in top laboratories.^32^ Therefore, as will be demonstrated, it is difficult to ascertain exactly where Menten was at any given point in time. However, during portions of 1910–1912, Menten worked as a research fellow in the laboratory of George Washington Crile (1864–1943) at Western Reserve University in Cleveland, OH. During 1911, Menten completed her MD at the UofT (NB, while numerous Canadian women had previously graduated with bachelor of medicine degrees, as had Menten, she was one of the first women to complete the research-intensive doctor of medicine degree at UofT). Menten returned to Cleveland in 1911–1912 where she worked on the control of acid-base response during anesthesia and other topics before going to Berlin to work with biochemist Leonor Michaelis (1875–1949). Her temporarily departure from Crile’s laboratory was because Michaelis was an internationally acclaimed expert on pH and buffers.^45^

Sometime before September 1913,^46^ she retuned to Crile’s laboratory. In 1915–1916, she did cancer research at the Barnard Free Skin and Cancer Hospital in St Louis^13^ while simultaneously pursuing a PhD in Physiological Chemistry at the University of Chicago. Her PhD was awarded on June 16, 1916 (personal communication, Andrea Twiss-Brooks, June 2, 2023). While her dissertation has generally been reported to address the effects of adrenaline on hemoglobin,^45^ this is actually incorrect; nevertheless, she did publish a sole-authored paper entitled “The action of adrenalin on the blood” in the *American Journal of Physiology* in September 1917; this paper was submitted “from the Ryerson Physical Laboratory and the Hull Laboratory of Biochemistry of the University of Chicago” on July 3, 1917.^47^ It should be noted that Menten was working in Pittsburgh at the time of submission. She also published at least one other paper from work done in Chicago.^48^

Her dissertation, which has been recently digitized by the University of Chicago, is entitled “The alkalinity of the blood in malignancy and other pathological conditions together with observations on the relation of the alkalinity of the blood to barometric pressure.”^49^ Her PhD supervisor was Albert Prescott Matthews, PhD (1871–1958), professor and chair of the Department of Physiological Chemistry.^50^ Menten’s dissertation was actually reprinted from a sole-authored paper of the same title that she published “from the Department of Pathology of the Barnard Free Skin and Cancer Hospital, St. Louis, Missouri” in April 1917.^51^ This paper clearly built on her work in Crile’s lab. In it, she acknowledges “Dr. George M. Smith, the former Director of the [Barnard] Hospital and Laboratories, for his generous support and assistance. Without his aid, much of the work could not be done.” She also acknowledges two residents who drew patient blood samples for her research and “Professor [of Physiology and soon to be Nobel laureate Joseph] Erlanger. . . for the loan of apparatus.” (p. 210).^51^ So, strangely, her University of Chicago dissertation research was performed in Smith’s laboratory in St. Louis. It should also be noted that Smith was a founding editor of the *Journal of Cancer Research*,^52^ where her dissertation was published. In this publication, Menten acknowledges that “her sincere thanks are due to Prof. A. P. Mathews of the University of Chicago for suggestions and criticisms in the preparation of the manuscript.” (p. 210).^51^ All of this appears to have been another example of an informal arrangement as the Washington University in St. Louis Becker Archives lacks any extant hospital or university records documenting Menten’s time in St. Louis (personal communication, Philip Skroska, June 2, 2023). Menten reported to her sorority sisters that she was “doing research work in physiological chemistry at the University of Chicago” in January 1915^53^ and that she was “doing research in St. Louis in December 1915.”^54^ After these 2 reports, Menten appears to have “outgrown” reporting her location to her Theta sorority sisters, which, had she not spent her next 34 years in Pittsburgh, would have made her harder to track.

As noted earlier, she became the pathologist at the Elizabeth Steel Magee Hospital in 1916 and she accepted a junior faculty position in the Pitt pathology department in 1918. She was a pediatric pathologist at Children’s Hospital of Pittsburgh from 1926 to 1950.

After retiring from Pitt at the age of 71 years (Figure 3(b)), she ended her research career back in Canada as a research fellow at the British Columbia Medical Research Institute (1951–1954); here she spent several years measuring nucleic acid content in samples for a Dr. M. Willms.^55-60^

To better understand her important contributions to surgery, biochemistry, and pathology, her accomplishments in Cleveland, Berlin, and Pittsburgh merit in depth analysis. Her work in these locations also provide insights into her personality at various stages of her career.

### Menten’s Work in Cleveland

Prior to the late 19^th^ century, the ideal surgeon was bold, brilliant, and, in the absence of anesthesia to control pain, worked quickly; by the early 20^th^ century, animal studies were showing that “physiological” surgery, focused on meticulous technique, painstaking attention to hemostasis, gentle tissue handling, minimization of tissue hypoxia, and attention to maintaining a normal acid-base equilibrium provided better results.^61,62^ William S. Halsted (1852–1922), chair of surgery at Johns Hopkins led this transformation in North America.^63-66^ During the first few decades of the 20^th^ century, surgeon George Washington Crile (1864–1943), a co-founder of the Cleveland Clinic, performed a transformative series of studies exploring this concept.^62,67^ Menten’s breadth of skills were highly complimentary to those of Crile and his surgical colleagues, and her willingness to travel to learn new ones, transformed some of this important work.

Menten worked sporadically (i.e., portions of 1910–1912 and 1913–1914) with Crile at Western Reserve. While it is fair to say that she pushed Crile’s physiological surgery research in new directions, his 2-volume autobiography, edited and published posthumously by his wife Grace Crile, mentions Menten in only 1 sentence: “For several years, in association with Dr. M.L. Menten. . . [and three others], I had been engaged in a research on hydrogen-ion concentration in the blood – the H-ion index being a measure of the acidity of the blood – in normal and pathologic states.” (p. 269).^67^ The Criles then summarize this fundamental research as follows:

Among the many clarifying results that these studies presented was the observation that the hydrogen-ion concentration of the blood steadily increased during anesthesia and at the exact time that all of the buffer substances in the blood were overcome and the blood became acid, death inevitably occurred. This finding disclosed a fundamental fact in the living – it disclosed that protoplasm ceases to function when it becomes preponderantly acid or preponderantly alkaline (p. 269).^67^

Most of the studies Menten worked on in Crile’s laboratory are published solely under Crile’s name, and she is simply acknowledged. She is regularly mentioned, sometimes very prominently, in many of his important publications and presentations to surgical conferences after 1912.^68-70^ For instance, in the opening to a 1918 paper entitled “The value and limitations of laboratory studies of acidosis in surgery” Crile credits the initial studies in 1912 in his laboratory investigating the relationship between acidosis and surgical shock to “Dr. M.L. Menten” and then lists the others who “later continued” it. He further notes:

In the work in collaboration with Doctor Menten, who had worked for a year with Michaelis and used his gas chain method, we found that the H-ion concentration of the blood was increased: (1) During intense fear; (2) during intense rage; (3) during extreme exertion; (4) during inhalation anesthesia; (5) in surgical shock; (6) in hemorrhage; (7) in asphyxia; . . . (11) near the point of dissolution whatever the cause of death. (p. 457)^70^

In fact Menten’s work played a supportive role in Crile’s re-definition of surgical shock as well as his approach to the “anociated operation” (contemporary surgical writers did not like this name and renamed this the “shockless operation) (p. 165).^62^ Crile notes that “the factors increasing acid by-products. . . should as far as possible be controlled by the surgeon. The *anociated* operation minimizes these injuring factors (p. 263).”^69^ In Crile’s gifted hands, operative mortality dropped from 6.2% to 1.7% after implementing his new protocols (p. 167).^63^ Crile published at least 2 surgical textbooks on *Anoci-Association*,^71,72^ a concept that is still occasionally cited in the pain literature 100 years later,^73,74^ Crile was obviously very excited about Menten’s data; he even credits her at the beginning of a talk on the physiological effects of emotions given to the American Philosophical Society.^75^ Crile’s data suggested that the simultaneous use of both local and general anesthesia promoted patient survival by preventing pain signals from reaching the brain. Psychologists were generally less enthusiastic about Crile’s research crossing into their arena.^62^

Crile linked his work on anoci-association to Charles Darwin’s concept of natural selection via a process he named “phylogenetic association” and, in 1913, to Walter B. Cannon’s work on the survival benefits of adrenaline secretion in response to fear and pain via a process Crile named the “kinetic system.” While all of this is beyond the scope of the current paper, it is described in detail elsewhere (pp. 155–187).^62^ Menten brought biochemical expertise to Crile’s laboratory at the time these novel concepts were being developed.

Menten not being named as a co-author is not evidence of sexism, as it was not uncommon for prominent surgeons to treat male assistants and junior male colleagues the same way.^65^ It was a symbiotic relationship that was transformative to both. Working with Crile launched Menten’s career; she also co-authored at least 2 papers with him^76,77^ and published a sole-authored paper entitled “The relations of potassium salts and other substances to local anesthesia of nerves” from his laboratory using techniques she learned from Macallum.^78^

However, 1 study, initiated by Menten in mid 1912, at most a few months before traveling to Germany, deserves particular mention as it provides unparalleled insights into her charm and demeanor while equally documenting her resourcefulness, tenaciousness, work ethic, and physical toughness. The amazing story of Maud Menten’s field work with legendary shark hunter Russell J. Coles (1865–1928) has been meticulously researched by coastal North Carolina historian David Cecelski.^79^ According to Ceceliski:

Crile had a longtime interest in the physiological changes that stress, trauma and exhaustion cause in the human body. As one component of that research, he did a series of animal studies involving the effects of exhaustion on nerve cells. In that research, he examined the nerve cells of animals that had been driven to exhaustion as either part of their normal life cycle or because of human intervention – foxes that hunters have chased. . . or salmon after that had migrated upriver to spawn. . .. With Menten’s help, Crile was able to study the effects of exhaustion on the nerve cells of the Brazilian electric ray. . . [which use an] electric charge to stun prey and to protect themselves.^79^

Menten began by writing Barton Bean (1860–1947), the Smithsonian’s assistant curator for fish, who recommended she contact Coles, who had been observing and collecting this species annually off Cape Lookout, NC. Based upon his soon to be published data,^80^ this ray species migrated into the area annually on about June 29 and left the area before July 4. Armed with the knowledge of this narrow window of opportunity, Menten quickly mobilized and arrived on June 28, 1912, and then she and Coles began collecting electric rays the following morning. Cecelski described Menten as “indefatigable” and “a force of nature.” His analysis is based largely upon reading correspondence between Cole and Bean. Cole spoke of Menten in glowing terms noting how they collected rays all day and worked in the laboratory at night. According to Cecelski:

To say the least, Coles was enthralled. His letters and diaries generally show a predatory view of women – as objects to be hunted, conquered and then cast aside, not wholly unlike his attitude towards sharks. Not true with Menten. In speaking of the week of hospitality and energy he devoted to Menten, he referred only to her work and her ideas. On July 24, 1912, he wrote Bean. . . “I will feel well repaid in being able to help make Dr. Menten’s great work. . . a success. . .. Dr. Menten is unquestionably the most wonderful human being in the world”^79^

Coles is a fascinating but challenging historical figure; he had an inquisitive mind, a zest for life, and, in addition to being a womanizer, strong predilections for activities now uniformly considered inappropriate. He made his fortune in the tobacco industry and then transitioned into an adventurist “big game fisherman” based at Morehead City, NC. During the summers, he spent weeks on his large “house boat” with a crew of hired fishermen hunting sharks, devil fish, and rays up and down the coast. While his crew trawled and seined, Cole’s preferred method was to venture out along a shoal miles off shore in a lone small whaleboat, and though “far from young,” stand on its prow “with his harpoon, made by his own hands in his own forge, and go after. . . [sharks] until he was exhausted and the sea blood red.”^81,82^ While he profited from the shark steak and shark leather industries, he was simultaneously scientific-minded; he collected specimens for natural history museums, published behavioral observations, and was recognized as a leading cartilaginous fish ichthyologist. He and Teddy Roosevelt became long-time, close fishing buddies and were simultaneously awarded honorary DSc degrees from Trinity College in 1918.^81,82^ However, one cannot help but cringe imagining the juxtaposition of these 2 people in a cramped work environment, as the contrast between Menten and Coles could not be more striking! Yet, they worked productively together; she clearly impressed Coles and may have forced him to at least reconsider some of his sexist stereotypic beliefs about women.

Crile published a 6-part series of papers entitled ”Studies in exhaustion” in the *Archives of Surgery* between 1920 and 1924; the paper describing Menten’s work with Brazilian rays appeared in July 1921,^83^ 9 years after Menten’s field and laboratory work at Cape Lookout. Menten was acknowledged but not a co-author.

## Menten’s Brief Time in Berlin

Shortly after returning to Cleveland from North Carolina, Menten traveled by boat to Europe, only months after the sinking of the Titanic, and worked in Berlin for about a year in the laboratory of world-renowned biochemist Leonor Michaelis; the precise dates are unknown. This in itself was another adventure as she was a 33-year-old, single, anglophone woman working as a research assistant, likely without pay. Although Menten could apparently read scientific articles in German prior to her arrival, she needed to quickly learn to speak and write. Furthermore, laboratory dynamics may have been awkward as Michaelis was only 4 years older than Menten, an orthodox Jew, and married.

Michaelis’s personal situation was suboptimal for scientific productivity. He had completed his research training in top laboratories, including working with future Nobel laureate Paul Ehrlich (1854–1915), more than a decade earlier but decided to train in clinical medicine because professorial positions in biochemistry were not likely available to him because of antisemitism. In fact, Michaelis, though internationally renowned, never held an academic position in Germany. At the time of Menten’s arrival, he had only the equivalent of an unpaid clinical professor appointment. Therefore, he conducted part-time research while supporting his family. as director of laboratories at the Berlin Municipal Hospital (1906–1922), where he functioned as a bacteriologist. However, he established a small research laboratory within.^45,84-87^

Because Michaelis’s research was mostly focused on acid-base chemistry, it has been speculated that Crile “advised her to learn about pH and buffers from the world’s leading expert. . .” (p. 46).^45^ However, it seems unlikely this was entirely Crile’s idea, as Menten had begun citing Michaelis’s earlier work on fat stains and his textbook on Farbstoffchemie while a student assistant working in Macallum’s laboratory. Furthermore, Menten had a reputation for making her own decisions.^1,14^

Soon, Menten was also studying enzyme kinetics with Michaelis. Albert Gjedde summarizes this work succinctly as follows:

Maude Menten assumed a role in the studies of the velocity of the invertase (Invertin) reaction with sucrose (Saccharose) to form glucose and fructose (Lävulose), a reaction known as inversion because it is accompanied by a change of optical rotation from dextrorotation of sucrose and glucose toward levorotation of fructose (hence the German name for fructose). In the studies, the reaction velocity was measured by polarimetry and expressed in units of net rotation change per minute. Most of the theory had been presented in previous papers by Michaelis and other co-workers, and Michaelis’ name was attached to a “graphical” method of determination of affinity constants that required only some modification to lead to the complete Michaelis-Menten equation. The joint paper reporting the quantification of the invertase reaction was submitted February 4, 1913. (p. 244)^84^

Menten had been working in Berlin for at most 6 months at the time of submission of their ground-breaking paper,^88^ and Giedde questions whether the entire study could have been completed by her:

Judging on the basis of the number of experiments reported in the paper, it is unlikely that the experiments were initiated solely by Menten’s arrival, and her exact role remains to be determined. (p. 244)^84^

However, Giedde was clearly unaware of Menten’s tenacity and her well-documented tendency to work 18-hour days. Another source suggested that she likely did most of the work:

It is probably correct to write her results. . . because, although one cannot know exactly who did what in Michaelis’s laboratory a century ago, he was involved in so many different projects, with 94 publications in the 5 years before the First World War, that it is difficult to believe that he carried out many of the experiments himself. (p. 448)^85^

Regardless, Menten’s name is forever linked to this work. Since she had no financial support from Michaelis, some have speculated that she may have had a part-time position as a physician in his hospital.^45^ If so, this would have forced her to provide medical services in German, which is not entirely inconceivable because of her amazing linguistic abilities (see below). It is also possible that Crile provided some financial support since she would be returning to his laboratory with new expertise. Unfortunately, with the exception of Menten’s report to her sorority sisters that she was “studying in Berlin, Germany” in January 1913,^89^ the only extant documentation of her time there is her publication with Michaelis.^88^ Everything else is speculation.

Several histories have stressed that her byline under the title of the paper was “Miss Menten” rather than Dr. Menten.^1,45^ While it was commonplace then for young single or married female research assistants without doctoral degrees to be listed in a string of authors as either Miss or Mrs., the names of women authors with MD or PhD degrees were usually no longer defined using either of these sexist adjectives.^32^ Michaelis was likely responsible for adding this.

Likely by mid-1913, Menten was back in Cleveland. In 1922, during the post World War I financial crisis but before the overt rise of Nazism, Michaelis accepted a visiting professor position in Japan. Three years later, he relocated to Johns Hopkins University as a lecturer. In 1929, he was hired by Simon Flexner as a permanent member of the Rockefeller Institute, where he worked productively until he retired in 1941. He died in New York in 1949 at the age of 74.^85,87^

At the time of the centenary of the discovery, the original Michaelis-Menten paper^88^ was translated into English by Johnson and Grody^90^; the entire translation is available as an online Supplemental Material. They also reanalyzed the original data using modern computational methods and found “an unanticipated rigor and precision in the original publication (p. 8264).^90^ Their detailed analysis is beyond the scope of the current paper.

## Menten’s Long Career in Pittsburgh as a Clinician-Scientist Pediatric Pathologist (1916–1950)

Menten’s obituary in *Nature*^13^ was co-written by a Pitt colleague, Anna-Mary Carpenter, who was based in the pathology department at Children’s from 1938 to 1954^91^; therefore, it can be used to gain some firsthand insights into her career as a pediatric pathologist clinician-scientist. According to Carpenter and Aaron H. Stock, a Pitt professor of bacteriology and immunology who published at least 1 paper with Menten^92^ toward the beginning of his career:

Maud Menten was an avid research worker all of her life. In Pittsburgh, despite full schedules as a hospital pathologist and teacher of pathology, she continued to make noteworthy contributions to scientific literature. She was a versatile scientist as becomes a pathologist. . . [NB, I have removed a summary of her discoveries] Maud Menten was untiring in her efforts on behalf of sick children. She was an inspiring teacher who stimulated medical students, resident physicians and research associates to their best efforts. She will long be remembered by her associates for her keen mind, for a certain dignity of manner, for unobtrusive modesty, for her wit, and above all for her enthusiasm of research.^13^

It should be noted that Menten was Carpenter’s mentor, and they published at least 5 papers together.^93-97^

Another primary historical source is the brief but highly personal account in *Perspectives in Pediatric Pathology* by George H. Fetterman (1908–1988).^14,98^ They knew each other for over 2 decades, beginning with her teaching him as a medical student. According to Fetterman:

As to her clinical duties, Dr. Menten was at once surgical pathologist, postmortem pathologist, and hematologist. She was made aware of every puzzling or interesting case admitted to Children’s Hospital during her tenure. The pediatric residents flocked to the laboratory to consult her, and she was never too busy to listen. . . By working 18 hours per day, she discharged her obligations to education and patient care with substantial amounts of time left over for original research. She was full of ideas and highly critical for researchers who ran out of them. With her, it was a matter of, “what have you discovered recently?” In discussing the career of a world-renowned physician who had been awarded the Nobel Prize, Dr. Menten’s comment to me was “What has he done since?” Fortunately, she was more lenient with medical students than with Nobel Prize winners. (p. 6)^14^

Fetterman provides insights into her teaching and her temperament. He notes that she handled one-third of the teaching load for the second-year pathology course and that “the subjects Dr. Menten ‘drew’ for her lectures were, for the most part, ‘way out’ topics. In retrospect I believe she probably asked for them, but I’m sure she never asked for membership on a committee!. . .” (p. 6).^14^ He described her as “a dainty, personable, and striking woman” who “possessed a strong, brilliant mind” and noted that:

She may have seemed frail, but she was literally a mountain climber. She was also a warm and forgiving person. When she took a student to task, the words of correction were usually accompanied by a smile. If she shook her head and laughed at such times, the student knew that he or she had really pulled a dandy. (p. 6)^14^

Fetterman noted she was strong-willed and could be a handful to manage, while still exuding a certain charm:

Dr. Menten reported not only to the Professor and Chairman of Pathology but also to the Medical Director of Children’s Hospital, who was Professor and Chairman of Pediatrics. There were occasions, I am sure, when both chairmen reported to Dr. Menten! With her personality, of course, she would have made that easy for them to do. (p. 6)^14^

Fetterman.^98^ replaced Menten as director of laboratories at Children’s Hospital when she retired in 1950. His first pediatric pathology publication was a series of 3 cases of advanced coronary artery sclerosis in infants published with Menten.^99^

Rebecca Skloot noted that Menten set high standards and could become animated when she believed it might help:

She wasn’t easy to please. . . To a laboratory full of scientists who she thought needed to work harder, or in a different direction, she would let loose with a tirade, and then fasten her hat firm as she stormed out the door, saying, “I’ve stirred them up and so now I can go. (p. 21)^1^

She was also a woman of diverse interests. She reportedly had amazing linguistic skills, In addition to her afore-mentioned fluency in English, German, and Halq´eméylem, she could also read and speak Russian, French, and Italian. She enjoyed traveling and outdoor activities (astronomy, swimming, hiking, and camping), and she once participated in an arctic expedition.

In stark contrast, she also had a “passion. . . for tea time, which she was known to observe with homemade scones and Scottish shortbread on Royal Crown Derby china.” (p. 21)^1^ According to Fetterman:

Dr. Menten was a music lover; she was both a listener and a participant [reportedly an accomplished clarinetist^35^]. She also loved to paint and painted well. A number of her oils appeared in highly competitive exhibits. . . In fact, one of my most valued possessions is a still life she painted.” (p. 6)^14^

A search of Pittsburgh newspapers document that some of her paintings received local acclaim. She was1 of ~26 local artists invited to display 2 paintings at an exhibition of paintings by Pittsburgh artists at the Carnegie Institute on both June 6-July, 1935 and June 8-July 31, 1937. In the later exhibit, Menten’s “Tulips” received special favorable mention in the *Post-Gazette* coverage.^100-102^ She also promoted art, such as when world-renowned German-American abstract artist Jans Hofmann (1880–1966) held a solo exhibit in Pittsburgh. It was sponsored by 9 of his former students, including “Dr. Maud Menten.”^103^

While Menten appeared to be good at everything, her driving skills, or more accurately lack there of, were legendary. According to Fetterman:

She lived in an apartment in Shadyside, an avant garde residential district. . . An independent woman, she owned a model T Ford; it was a prized possession. Dr. Menten was an early practitioner of the “jackrabbit start.” Once the engine was chugging, anyone absorbed in good-byes was well advised to jump clear. Despite many dire predictions, she never really accumulated any traffic violations. (p. 6)^14^

Skloot described her driving skills with a little more flair and then linked her driving to her personality:

Through the ice of winters and the balmy warmth of summers from 1918 to 1950, Maud Menten lurched through Shadyside and Oakland in her Model T Ford. After her jackrabbit starts, she would settle behind a wheel far wider and taller than she was, leaning slightly forward, wearing her Paris hats, blue dresses with stained-glass hues, and Buster Brown shoes. She never knew exactly which pedal to push when. . . She wasn’t sure so she would push them all. Folks said she made up with enthusiasm and quick starts what she lacked in driving skill and they knew to stay out of her way. On the road it was for fear of losing their lives, but elsewhere, because they knew she was unstoppable. Driving her Model T was about the only thing Menten couldn’t do. And if anyone tried to talk her out of anything. . . Menten, whose petite frame and sea-blue eyes projected only tenderness, would smile sweetly and keep right on doing it her way. (p. 19)^1^

Sadly, there was one other thing Menten could not do and that was timely promotion to full professor and perhaps, also, to department head. Barbara Paull noted in her history of Pitt Medical School:

Regrettably, Menten’s accomplishments were insufficiently regarded by the University of Pittsburgh School of Medicine. She did not attain the rank of full professor until 1949, the year of her retirement, after thirty-one years on the faculty. A friend and associate, Dr. Ann-Mary Carpenter. . . wrote that Dr. Menten, who “wanted full professorship so acutely,” was “deeply hurt” when another pathologist was named department chairman. “In terms of women’s lib, Dr. Menten could have written volumes,” Carpenter said. “Tis amazing that women who did work hard, had knowledge and achievement were not rewarded. My own wish is that she could have been recognized, but apparently she was too far ahead of her time.” . . . perhaps because of the women’s movement, her accomplishments have recently sparked new interest. . . Yet, according to her friend, Menten “would be livid about the interest and honor coming so late when she was given no recognition during her career.” (p. 107)^104^

With unparalleled irony, Pitt’s current chair’s full title is the “Maud Menten Distinguished Professor and Chair of Pathology.”

## Menten’s Most Important Experimental Pathology Contributions in Pittsburgh

Menten’s earliest experimental pathology studies at Pitt were responsible for her rapid promotion from demonstrator (1918) to assistant professor in 1923. These studies examined the mechanism of conversion of colorless chemical compounds to colored ones by oxidation^105,106^ and the nephrotoxic effects of mercuric chloride in kidney.^107,108^ In 1925, she and Helen M. Manning observed rabbits infected with Salmonella become hyperglycemic and this was associated with bacterial endotoxin depleting hepatic glycogen stores.^109^ This important discovery and her other work on glycemic effects of bacterial and biologic products^110,111^ clinched her promotion to associate professor and her appointment as pathologist at Children’s Hospital, both in 1926.

Not surprisingly, while refocusing on her new pediatric pathology clinical roles, her experimental pathology research productivity waned and lacked focus. In the wake of the recent prominence of fish in the discovery of insulin,^112^ her earlier work on the effects of asphyxia, and her recent work on the glycemia effects of Salmonella, she dedicated some time to studying glycemia in normal and asphyxiated cod, sculpin, and pollock.^113^ According to the “news of the classes” section of the July1928 edition of the *University of Chicago Magazine*, she spent the summer of 1928 doing research at the US Fisheries Station at Booth Bay Harbour, ME.^114^ She must have been particularly pleased, now being in her late 40s, to report that she could still perform such rigorous field work, as this represents the only project she ever reported in her PhD alma mater’s class news. Perhaps this was reported specifically for the benefit of her PhD supervisor, who regularly spent his summers working at Wood’s Hole Marine Station, or maybe just because famous physiologists of the day were fascinated with fish “islet organs.”^112^

In the 1930s, Menten completed a series of strange studies with Charles Glen King (1896–1988), an internationally-acclaimed Pitt nutritional biochemist and co-discoverer of vitamin C,^115^ showing that guinea pigs maintained on vitamin C deficient diets, not severe enough to induce scurvy, have diverse adverse effects on physiological processes in animals receiving small doses of diphtheria toxins^116^; she observed that deficient animals develop various pathological lesions including hyperplastic arteriosclerosis and hyperglycemia associated with hydropic degeneration of the pancreatic islets.^117,118^ The doldrums of this transition period was perhaps unavoidable as she was expending most of her immunological and chemical skills in the clinical laboratory purifying the toxin responsible for scarlet fever (see below). Not surprisingly, she convinced King to collaborate on purification of bacterial toxins.^119^

By the early 1940s, and with her scarlet fever work behind her, Menten was beginning to master her complex clinician-scientist role. The quality of her scientific work peaked in 1944 when she published 2 of her most important technique papers. In one, Menten and 2 colleagues used sedimentation and electrophoresis to distinguish fetal from adult carbonylhemoglobin^120^; According to her nature obituary: “this [was] the first use of electrophoretic mobility to determine differences in human haemoglobin, antedating [by five years] Linus Pauling,” who more famously used the same basic approach to study hemoglobin in sickle cell disease.^13^

Also in 1944, Menten et al. published a method paper in which they used an azo dye to detect alkaline phosphatase in renal tissue; the ingenious key to the new technique was detection of the alcoholic product of hydrolysis of the phosphate ester substrate.^121^ As noted in her *Nature* obituary: “This piece of work was called enthusiastically “a stroke of genius” by A.G. Pearse in the first edition of his book on *Histochemistry* because it opened up a field of enzyme histochemistry. . .^13^

Menten had a prior interest in toxicological pathology. Having studied the adverse effects of drugs on the kidney dating back to her mercuric chloride studies,^107,108^ she now used her alkaline phosphatase technique to study alloxan renal toxicity.^122^ Next she focused a series of studies on bone marrow toxicity associated with sulfonamide therapy.^123-125^

Menten’s productivity during the 1940s was extraordinary; she was promoted to full professor in 1949 and then retired a year later.

## Menten’s Pediatric Pathology Contributions in Pittsburgh

Menten was known locally for her work focused on the prevention of scarlet fever in the 1930s and 1940s. Newspaper coverage noted that scarlet fever death rates had plummeted “from 44.7 per 100,000 population in 1892 to 0.045 deaths in 1941,” and that this was due to preliminary work in Chicago that had been expanded and improved in Pittsburgh. Acc-ording to *The Pittsburgh Press* on May 19, 1942:

Over 20 years ago, University of Chicago research revealed the cause of the disease to be streptococcic germs found in the throats of scarlet fever patients. Streptococci obtained from such patients were grown in a fluid and from this a substance containing the toxin was separated. And now, as a result of five-year research in the Children’s Hospital laboratories, scarlet fever toxin has been purified to obtain the maximum immunization with the least harmful effects. The purified toxin is 10,000 times more concentrated than any obtained previously. For five years immunizations have been carried out. . . Schools in this district where immunization has been carried out have been virtually free of scarlet fever. . . Research has been under the direction. . . of Dr. Maud L. Menten. . .^126^

In an earlier article, this one written for the lay public by Menten, she explains to the readership of the *Pittsburgh Post-Gazette* how a simple skin test, the Dick test designed by husband and wife physicians George and Gladys Dick (1881–1967, and 1881–1963, respectively) of Chicago, can be used to determine which school children are or are not susceptible to developing scarlet fever and then describes how susceptible children can be made immune by a series of 3–5 injections of toxin at increasing doses.^127^

Her clinical research publications from Children’s include chemistry,^128,129^ autopsy or surgical pathology case repo-rts,^99,129-134^ and several case series.^135,136^ The most important of these was her description of how treatment with new antibacterial agents altered the histopathologic findings in childhood pneumonias.^136^ Menten also had a long-standing interest in the bacteriology of childhood pneumonias.^137^ However, as described above, her most important papers published while working as a pediatric pathologist were in the field of experimental pathology.

### Menten’s Promotion of Women in Medical Science

With the exception of the new field of bacteriology, women were generally not very welcome in biomedical sciences at the beginning of the 20^th^ century.^32^ Nevertheless, Menten spoke at venues promoting women in science. For instance, as early as 1923, she participated in a roundtable discussion with 4 other women entitled “How wise is it for a woman to choose a life career in a scientific field” and then gave an hour-long address entitled “The medical research worker.”^138^

She was an occasional speaker at the Women’s Medical Society of Pittsburgh meetings and more frequently at the local Sigma Sigma Epsilon women’s medical fraternity at Pitt, also serving as the group’s faculty advisor, as well as at the Zeta Phi national women’s medical fraternity.^139^ She mentored women trainees such as “Miss Iola Graham” who she supervised for a year-long project on scarlet fever serum research.^140^

As alluded to above, one of Menten’s mentees achieved a highly distinguished career. Anna-Mary Carpenter received her AB from Geneve College (1936) and then her MA (1937) and PhD (1940) in botany from Pitt. She then worked as Menten’s research associate from 1942 until Menten’s retirement.^93-97^ Carpenter later left Pitt, graduated with a MD from University of Minnesota in 1958, and stayed on in Minneapolis rising to the rank of professor of histology in 1963. She became a superstar in histochemistry and morphometrics, was awarded an honorary DSc by Geneva College in 1968, and was also awarded a Pioneer’s Award by the International Federation of Societies of Histochemistry and Cytochemistry.^141^ Her work on the anatomy of the middle and inner ear was also internationally acclaimed.^91^

Beginning in 1927, Menten was active in the American Association of University Women’s multi-year drive to endow 25 fellowships at $40,000/each. She helped the Pittsburgh branch raise $1500 for the national effort and also helped with the branch’s long-standing support of a summer fellowship at Bryn Mawr College.^142,143^

## Fund-Raising for the Children’s Hospital

Menten was an asset when Children’s Hospital was attempting to educate the lay public or secure donations. For instance, as previously mentioned, she published a paper in 1939 directed at laypersons entitled “Scarlet fever and its prevention” for *Keeping Healthy*, a “series of articles by prominent authorities in medicine and public health prepared especially for the *Post-Gazette* under the auspices of the General Health Council, a Community Fund Agency.” After providing a primer on the science behind the process, she confidently concluded: “With full cooperation of parents and physicians it should be possible, by means of this immunization process, practically to eliminate scarlet fever from our midst.”^127^ However, immunization against scarlet fever was soon superseded by the advent of penicillin in the 1940s. Speaking at “Children’s Hospital Day” on March 30, 1949, Menten explained the differences between bacterial and viral pneumonias and why the “wonder drug” was ineffective against the latter. She explained that viral pneumonia was not new but had previously been masked by bacterial pneumonia.^144^

Menten was featured during the “buy a Children’s Hospital flower” fund-raising campaign during World War II; the *Pittsburgh Press* published an uplifting article entitled “Another Maud”

Dr. Menten. . . is in charge of the Children’s Hospital remarkable laboratory. Here in her High Tower (really only a few bare walls with test tubes and small animals awaiting their moment of Ultimate Usefulness), Dr. Menten is completing her research on scarlet fever immunization, on the development of Blood Bank Plasma, and innumerable other scientific explorations. I write of her today, instead of the hundreds of more dramatic human interest stories there to be found, because it seems to me that she exemplifies the greatest service to which the human intelligence can be directed in these days of violence and destruction. Unhesitatingly, I would place her along side the other Maud, Maud Slye, whose experimentations on mice in determining the heredity persuasions of cancer have been one of the major contributions to scientific research [NB, Maud Slye was a contemporary experimental pathologist at University of Chicago^32^ whose career had been much better publicized than that of Menten]. It is well to remember these cloistered priestesses, who even now, with the world rocking around them, have so trained their powers of concentration and curiosity that these are able to proceed under great handicaps in their search for new means to save man from his own folly.^145^

## A Final Mystery—Menten’s Middle Name

Maud Menten was not given a middle name at birth, but, for unknown reasons, she added one, “Lenora” c1900 and then later changed the spelling to “Leonora.”^16^ It has been speculated that she may have picked the new spelling because of its similarity to Leonor Michaelis, but there is no way to confirm this.^45,85^ Her first publication^37^ with Macallum (i.e., 6 years before meeting Michaelis) lists her as “Miss M.L. Menten, B.A.,” and so this is consistent with her having already picked a middle name but is not informative as to its spelling. Most of her subsequent publications provide only a middle initial “L” and so are not helpful. To further confuse matters, her middle name is spelled “Lenore” in her *American Men of Science* entry.^28^ Her 1916 PhD dissertation spells her middle name “Leonora.”^49^

## Menten’s End of Life

Menten never married or had children, but she appears to have been a dutiful daughter, who helped care for her mother, Emma, who out-lived her husband by almost 30 years; Emma moved to Toronto by 1905 to be near Maud and some other family members; she also joined Maud in Pittsburgh, likely before 1924.^35^ Emma’s obituary reported that she died at home on December 16, 1932.^146^ Emma’s will was drawn up in Pittsburgh.^35^

After retirement from Pitt in 1950, Menten moved to Vancouver, and then, suffering from debilitating arthritis, relocated to Ontario 6 years before her death. Menten died on July 17, 1960 in Leamington, Ontario; her body was cremated, and her ashes were interred in the Menten family plot at Little Mountain Cemetery in Chilliwack, BC.^16,31,35^

A former colleague, upon hearing of her death, commented: “She did not waste away, she used herself up.” (p. 21)^1^

## Menten’s Eventual Recognition

As should be clear by now, reviewing Menten’s career is difficult because of her tendency to multitask. She spent short periods of time working in diverse places, some of which are impossible to document except by citing the publications arising from her work at these centers. Sadly, there is nothing particularly unusual about this, as other women working in other North American academic pathology laboratories during the early decades of the 20^th^ century are equally difficult to document; unlike men, it was apparently not uncommon for women working in pathology laboratories to do so as unpaid volunteer researchers, making it impossible to find employment records.^32^ What perhaps is more unusual is that, unlike some other early 20^th^ century women in pathology, Maud Menten’s family was not wealthy or influential, and so it is unclear how she supported herself prior to her first real job in Pittsburgh. Because her career predated *Index Medicus* and because *IndexCat* is hardly encyclopedic, it is not possible to determine how many papers she published during her career; some have estimated up to perhaps 100.

**Figure 4.**
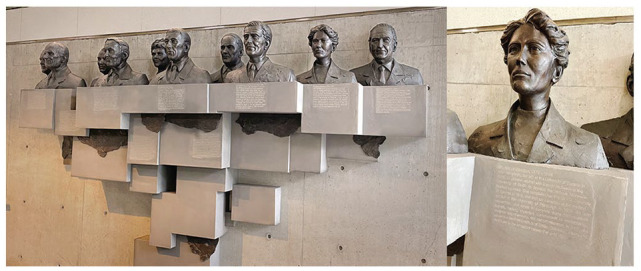
On the Shoulders of Giants at the Donnelly Centre of Cellular and Biomolecular Research at the University of Toronto. Stainless steel, bronze, laser etching by Dam de Nogales. Its inscription identifies her as 1 of 10 giants of biomedical science who began their careers or established their reputation at UofT. Busts of all 10 co-honorees (left). Menten’s bust with inscription below (right). Unfortunately, her inscription erroneously states that she obtained her PhD at University of Toronto rather than the University of Chicago. Photo Credit: Christopher Rutty, PhD.

Thought relatively unknown during her lifetime, Maud Menten has received many posthumous honors, especially as she has been discovered in recent years. The province of Ontario and her alma mater, UofT, have honored her with plaques and a sculpture (Figure 4). Her long-time academic home, Pitt, has honored her with portraits, a lectureship, and a professorship. In 1998, she was posthumously inducted into the Canadian Medical Hall of Fame.^147^

Unfortunately, as noted by her friend and former colleague Carpenter, she might have enjoyed some of this recognition during her lifetime.^104^ Perhaps, the ultimate evidence that she went unnoticed is that I can find no evidence that she was ever awarded an honorary degree by any university or college, including schools which regularly honored contemporary female biomedical scientists with demonstrably lesser career contributions.^32^

**Figure 5.**
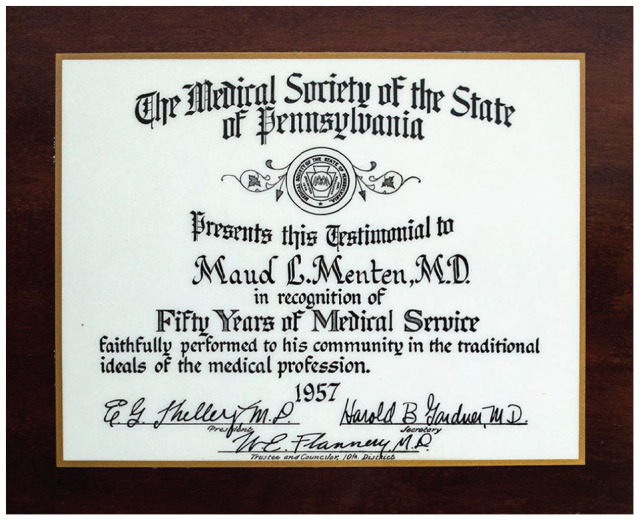
Maud Menten’s Medical Society of the State of Pennsylvania framed testimonial honoring her for 50 years of service since graduation from medical school. The wording of the plaque assumes that all recipients of such awards must be male. Credit: Kilby Historic Site. Artifact number KGSM1984.025.047.

Sadly, the only honor she did live to see was a plaque bestowed upon her by The Medical Society for the State of Pennsylvania on the 50^th^ anniversary of her medical school graduation with congratulations for “his” 50 years of medical service. This likely simultaneously annoyed and amused Menten; perhaps her sense of humor prompted her to have it framed (Figure 5).
